# Electrical integrity and week-long oscillation in fungal mycelia

**DOI:** 10.1038/s41598-024-66223-6

**Published:** 2024-07-06

**Authors:** Yu Fukasawa, Daisuke Akai, Takayuki Takehi, Yutaka Osada

**Affiliations:** 1 Laboratory of Forest Ecology, Graduate School of Agricultural Science, Tohoku University, 232-3 Yomogida, Naruko, Osaki, Miyagi 989-6711 Japan; 2 National Institute of Technology, Nagaoka College, 888 Nishi-Katakaimachi, Nagaoka, Niigata 940-0817 Japan; 3 Graduate School of Life Sciences, Tohoku University, 6-3 Aoba, Aramaki, Aoba-ku, Sendai, Miyagi 980-8578 Japan; 4 Present Address: Faculty of Engineering, Tohoku University, 6-6 Aoba, Aramaki, Aoba-ku, Sendai, Miyagi 980-8579 Japan

**Keywords:** Electrical potential, Foraging behavior, *Pholiota brunnescens*, Signal transport, Wood decay fungi, Fungal biology, Microbial ecology

## Abstract

The electrical potential of the mycelia of a cord-forming wood decay fungus, *Pholiota brunnescens*, was monitored for over 100 days on a plain agar plate during the colonization onto a wood bait. Causality analyses of the electrical potential at different locations of the mycelium revealed a clear and stable causal relationship with the directional flow of the electrical potential from the hyphae at the bait location to other parts of the mycelium. However, this causality disappeared after 60 days of incubation, coinciding with the onset of slow electrical oscillation at the bait location, which occurred over one week per oscillation cycle. We speculated that the hyphae that initially colonized the bait may act as a temporary activity center, which generates electrical signals to other parts of the mycelium, thereby facilitating the colonization of the entire mycelial body to the bait. The week-long electrical oscillation represents the longest oscillation period ever recorded in fungi and warrants further investigation to elucidate its function and stability in response to environmental stimuli.

## Introduction

Saprotrophic cord-forming basidiomycetes are widely distributed at the soil/litter interface on the forest floor^1^. They generate persistent linear cords, which are collections of parallel-aligned hyphae, that form extensive networks and search for dead plant material, such as leaf litter and dead wood, to acquire nutrients^1^. In addition, these fungi facilitate the transfer of carbon and nutrients across their mycelial networks^2^, thus playing a vital role in the cycling and redistribution of carbon and nutrients on the forest floor.

The behavior of cord-forming basidiomycetes has been extensively studied using soil microcosm systems^3,4^. In these systems, a wood block that has been colonized by cord-forming basidiomycete functions as an inoculum when placed on the surface of a compressed soil plate in a dish, which leads to mycelial growth out from the inoculum onto the soil. This mycelium then colonizes any newly encountered wood blocks (baits), forming a cord network between the inoculum and bait. Using this method, previous studies have identified a variety of “intelligent” behaviors in the fungal mycelia, such as decision-making based on nutrient status and past experiences^5–8^. For example, if a bait size exceeds a certain threshold, the mycelium will abandon the inoculum and migrate toward the bait, which indicates a decision-making process by the fungal mycelia that is dependent on the resource size^6^. Moreover, it was discovered that mycelia retain the memory of the bait’s direction even after the established cord connections were completely severed^6^. Similarly, a local disturbance of the mycelial network, which acts as a negative stimulus, also triggers memory formation and is related to decision-making in fungal mycelial behavior^5^. However, the mechanisms underlying these intelligent mycelial behaviors remain largely unexplored.

As an integrated system, the fungal mycelium transports materials and signals across its network, which is essential for the coordinated behavior of the mycelium^2^. Studies using radioactive tracers have demonstrated the simultaneous bi-directional translocation of nitrogen, phosphorus, and carbon across the mycelial network^9–14^. In mycorrhizal fungi, the transfer of signal molecules through the hyphae facilitates interplant communication via the fungal network^15,16^. Moreover, electrical signals, which are utilized by various organisms from unicellular aneural systems to multicellular neural systems for cognitive behavior^17^, may also play a critical role in signaling across the mycelial networks, thereby potentially offering a quicker alternative to liquid transport within the hyphae^16^. Previous studies have revealed that the mycelia of wood decay fungi, such as oyster mushrooms (*Pleurotus* spp.) and honey mushrooms (*Armillaria* spp.), exhibit spontaneous oscillation in electrical potential as well as action potential-like responses to environmental stimuli, including light, fire, salt, water, alcohol, carbon resources, and weight^18–24^. Furthermore, Adamatzky and colleagues observed that electrical action potential-like responses induced by environmental stimuli were identified not only in the focal fruit body but also in neighboring, undisturbed fruit bodies, which suggests the possibility of electrical signal transfer across fruit bodies via mycelial connections in *Pleurotus* spp*.*^20,21^. Similarly, Fukasawa et al.^25^ reported that the fruit bodies of an ectomycorrhizal fungus *Laccaria bicolor* exhibited electrical potential following a rainfall event, with causal relationships observed in their patterns across the neighboring fruit bodies. Nonetheless, this transfer of electrical signals across the entirety of the mycelia remains a subject of debate and ongoing research^26^.

In the present study, we measured the electrical potential at several points within a pure-cultured colony of a wood decay cord-forming basidiomycete *Pholiota brunnescens* on plain agar plates. The aim was to demonstrate the transfer of electrical signals related to stimuli (i.e., the addition of bait wood) across the mycelium by utilizing causality analysis of time-series data of electrical potentials that were recorded by electrodes attached at several points within a colony. *P. brunnescens* is a cosmopolitan species, and its mycelia have been revealed to possess a directional memory of wood bait^7^.

## Materials and methods

### Experimental setup

**Figure 1:**
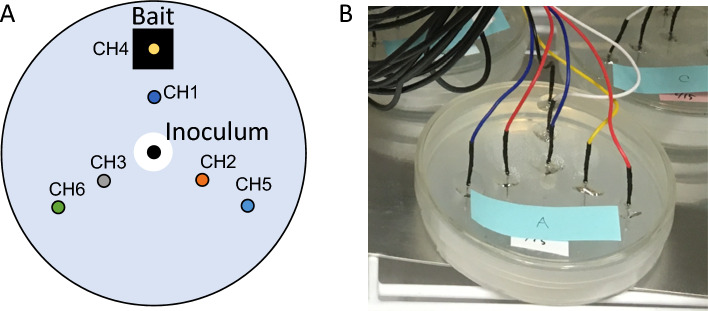
Schema (**A**) and picture (**B**) of a Petri dish equipped with the six subdermal electrodes, with a “base” electrode at the center (inoculum). The interval between electrodes was 1.5 cm each. A sterilized pine wood block (1 × 1 × 1 cm^3^) was placed onto the agar as bait at the location of electrode CH4.

We prepared Petri dishes (9 cm in diameter, 1.5 cm thick, Sanplatec Corp., Osaka, Japan) with seven subdermal needle electrodes (SPES MEDICAL SRL. Via Buccari 21, 16,153 Genova, Italy) that extended through the lid to the bottom of the dishes to measure the electrical potential of the mycelium on the water agar medium (Fig. 1). The electrodes that penetrated the lid were secured in place using UV resin (Kiyohara, Osaka, Japan). Before use, the dishes were sterilized with ethylene oxide gas at 55 ˚C for 2 h. A mycelial plug, which was cut out from an actively growing colony of *P. brunnescens* on a malt extract agar plate (0.5% malt extract, 1.5% agar, w/v; Nakalai Tesque, Kyoto, Japan), was placed at the center of the water agar media (1.5% agar, w v^−1^; Nakalai Tesque, Kyoto, Japan) in the electrode-equipped dish. The *P. brunnescens* strain (NITE Biological Resource Center, NBRC culture collection, strain #110175) was sourced from the dead wood of *Pinus densiflora*^27^. This fungus was selected based on the findings from our previous study, which demonstrated that this species possessed a directional memory of wood bait, thus suggesting an anticipated signal response throughout the mycelium when in proximity to the bait^7^.

A pine wood block (*P. densiflora*; 1 × 1 × 1 cm^3^) was sterilized three times with a one-day interval between each autoclaving and positioned on the water agar within the electrode dish, adjacent to one of the outermost electrodes (Fig. 1). To facilitate electrode penetration, a fine hole (3 mm in diameter) was drilled through the block before its sterilization. We prepared three replicate dishes with a wood block (bait), and an additional three dishes without a wood block (control). These dishes were then incubated at 20ºC in the dark.

### Recording of electrical potential

Recording of the electrical potential of the water agar plate was initiated within one day following the experiment setup. The electrical potential of the dishes was captured using a GRAPHTEC GL840-M Midi Logger (GRAPHTEC, Kanagawa, Japan). The electrical activity from the electrodes was logged every 10 min. This recording process continued for 104 days, during which the dishes were incubated. Throughout this incubation period, the margin of the colony was monitored to determine the timing at which the hyphal front reached the electrodes and the wood block. Note that the records contain several brank periods because of occasional power outages.

### Statistical analysis

Utilizing the time-series data of the electrical activity from the six electrode channels, we estimated the transfer entropy (*TE*)^28^ to identify potential causal relationships among the various points within a *P. brunnescens* colony. Transfer entropy, also known as a generalization of Granger causality, quantifies the directional flow of information between two entities in a nonparametric manner^29^. More precisely, it defines the flow of information from a causal variable (${x}_{t}$) to an effect variable (${y}_{t-p}$) with a causal time delay $p$ is defined as follows:

$$TE=\frac{1}{T}\sum_{t=1}^{T}\text{log}\frac{p\left({x}_{t}|{y}_{t-p},{x}_{t-\tau },{x}_{t-2\tau },\dots ,{x}_{t-E\tau }\right)}{p\left({x}_{t}|{x}_{t-\tau },{x}_{t-2\tau },\dots ,{x}_{t-E\tau }\right)}$$

where $E$ and $\tau$ represent the embedding dimension of the causal variable and the time interval required for the embedding time delay, respectively, and $T$ is the number of time points. In this study, we used the unified information-theoretic causality (UIC) method to estimate the *TE* and assess its significance^30^. To reduce the bias effects because of the small sample size, we applied effective transfer entropy (ETE), which adjusts for bias using surrogate data^31^.

Before conducting the UIC analysis, the time-series data of the electrical activity were first differenced to create a stationary time-series. In addition, we limited our analysis to the time-series data from the first 90 days to maintain system consistency. The embedding dimension ($E$) was selected to enhance the predictive accuracy of the simplex projection method^32^. The time interval ($\tau$) was set to the recording interval (i.e., 10 min) because the electrical activity of *P. brunnescens* is expected to change within a shorter time scale than the recorded interval. In the UIC analysis, we calculated the daily ETE rather than aggregating across the entire recorded 90 days, which enabled us to examine the temporal changes in the information flow. The analysis was conducted using a causal time delay ($p$) of 10 min, 720 min (12 h), 1440 min (1 day), and 7200 min (5 days) to assess the duration of causal influence. However, in the main text, we only present the results for the 1-day causal time delay because there was no qualitative difference in our findings across these intervals.

## Results

**Figure 2:**
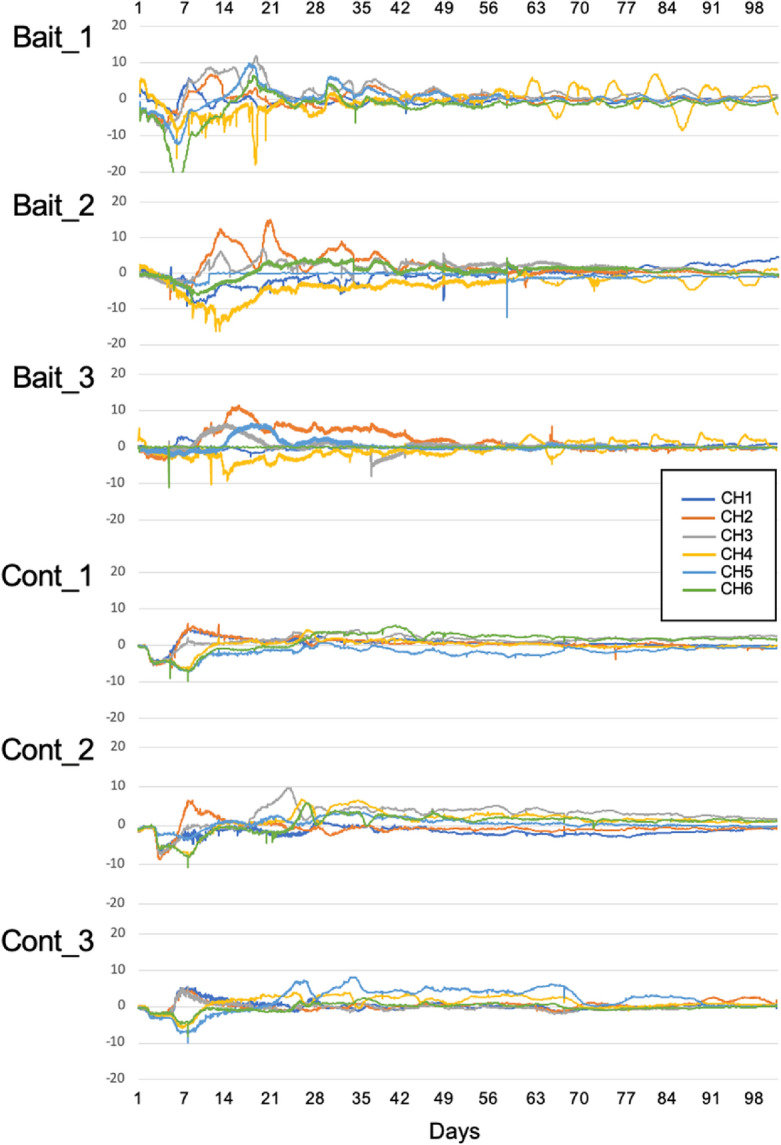
Electrical potential (mV) recorded on the electrodes in the three dishes with a pine wood block (Bait_replicate no.) and the three control dishes without wood bait (Cont_ replicate no.). Electrode CH4 in the bait dishes corresponds to the location of the wood bait.

The colony margin of *P. brunnescens* growing on plain agar reached the CH1–3 electrodes, located 15 mm from the center of the dish, on days 4–6, which coincided with an increase in the electrical potential of these electrodes (Fig. 2). Subsequently, the colony margin extended to the electrodes CH4–6, positioned 30 mm from the center of the dish, on the days 8–12, which corresponded with an increase in the electrical potential of these electrodes. The electrical potential at electrode CH4 in the baited dishes exhibited a distinct oscillation after day 63 (Fig. 2), with an oscillation period of approximately seven days.

**Figure 3:**
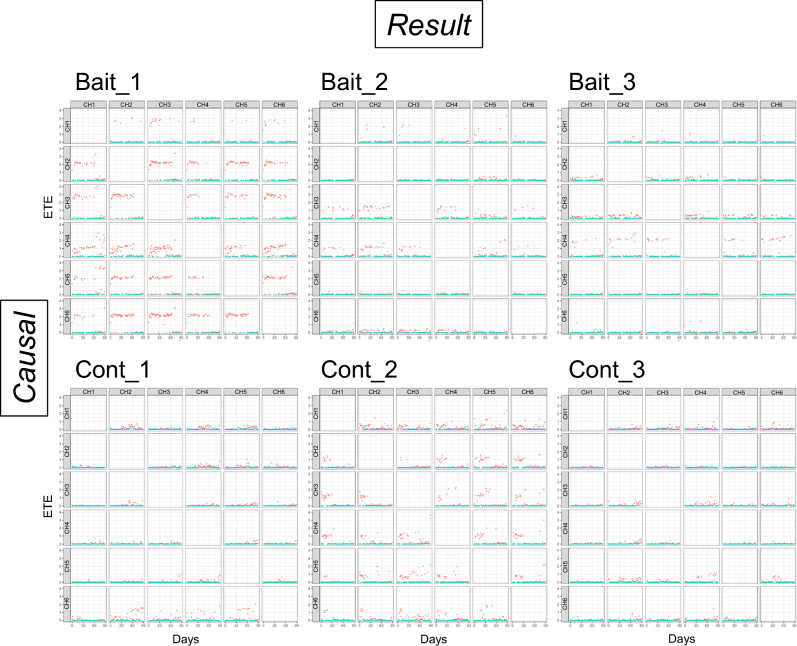
Effective transfer entropy (ETE) in the three replicate dishes with a pine wood block (Bait_ replicate no.) and the three control dishes without wood bait (Cont_ replicate no.) during the incubation period (days 0–90). The rows in the figure represent the *Causal* electrodes, and the columns represent the *Result* electrodes. A dot indicates the average ETE per day: red, significant (*p* < 0.05); blue, not significant. The causal time delay was set to be one day.

The UIC analysis revealed significant causal effects across the electrodes in both the baited and control dishes (Fig. 3). In the baited dishes, when the time delay in the causal effects was set to one day, the electrodes exhibited significantly large and stable ETE values, particularly during the first ca. 60 days, although the magnitude of the ETE values varied among the electrode pairs within a dish. Intriguingly, these stable ETEs in the baited dishes diminished after ca. 60 days in most electrode pairs, which coincides with the onset of obvious oscillations at electrode CH4 (i.e., the baited location). Furthermore, in the Bait_2 and Bait_3 dishes, large and stable ETE values were observed when electrode CH4 was used as the causal electrode, which suggests the potential directionality of the causal effects from electrode CH4 to the other electrodes. However, in the Bait_1 dish, significantly large and stable ETE values were observed in most electrode pairs in both directions and were not limited to the pairs involving electrode CH4. Similarly, in the Bait_2 dish, electrode CH3, in addition to electrode CH4, exhibited significantly large and stable ETE values with the other electrodes. These patterns in the causal effects between electrodes in the baited dishes were consistent across different time delays (10 min, 12 h, 5 days) (Supplementary Fig. S1). In contrast to the baited dishes, significantly large ETEs were observed in both directions for each pair of electrodes, without any discernible patterns during the incubation period (Fig. 2; Supplementary Fig. S1).

## Discussion

The synchronized timing of the colony growth around the electrodes and the increase in the electrical potentials at the focal electrodes suggest that fungal hyphae can alter the electricity of their surrounding environment. This is likely because of the physiological activities of hyphae, such as the transporting and exuding of nutrients and metabolites from hyphae to the agar^33^. For instance, wood decay fungi are known to secrete oxalic acid from their hyphae to reduce the environmental pH to their optimum range (pH 4–6)^34^, which could alter the electron density in the surrounding agar. The significant causal effects in the electrical potential across the electrodes suggest that the mycelium of *P. brunnescens* integrates its physiological activities and can transfer electrical potential across the different parts of the mycelial body. This is not surprising considering the notion of self-integration of the fungal mycelium proposed more than 20 years ago^35^ and recent studies that have reported the transfer of electrical potential in several wood decay fungi^21,33,36^.

A particularly exciting finding emerged from the dishes that contained wood bait, where the presence of the wood bait had a strong effect on the electrical patterns. First, a clear and long-period oscillation of electrical potential was observed at the baited position. Previous studies have reported a variety of oscillation time periods ranging from milliseconds to 20–40 s in action potential-like activities of the fungal mycelia^18,37,38^, to about 30 min in the extracellular electrical potential of *Pleurotus* sporocarps^20^, and 20–24 h (also referred to as circadian rhythms) in the electrical or physiological oscillation of fungal mycelia^19,39–41^. Because the measured electrical potential was not the action potential that occurred at the plasma membrane of the fungal cells but was likely attributable to the transportation and exudation of nutrients and metabolites^33^, it is reasonable that the oscillation period tended to be longer than those of previous reports. Nevertheless, the seven-day oscillation period observed in the present study was longer than any previously reported oscillation period. The mechanisms underlying this long-term oscillation period in the fungal mycelia are not known but may reflect the cycle of fungal wood decay activities. Previous studies have shown that wood decay by fungi can alter the electrical resistance of wood by secreting cations^42,43^. These effects might vary depending on the fungal species, corresponding to their types of wood decay (brown rot and white rot)^42^. Therefore, future research should compare these effects across various wood decay fungi.

Second, the strength of the significant causal effects remained stable throughout the duration of the incubation period in baited dishes, whereas the strength of the causal effects varied considerably in the control dishes. The strong and stable causal effects in the baited dishes suggested the emergence of strong and constant rhythms of electrical activities that were integrated across the entire mycelial network. Because this stability of the causal relationships was not observed in control dishes, resource availability might be the source of this electrical integrity in the mycelial systems. Similarly, in the oscillatory phenomena of nutrient transfer across the mycelium, the oscillation time period was not unified across the mycelium when the mycelium was still growing and searching for resources^39^. However, after the mycelium had grown sufficiently in the dish and colonized the resource(s), the oscillation became integrated across the mycelium^39^. Furthermore, fungal mycelia are able to synchronize their oscillation rhythms of nutrient transfer after the fusion of multiple but genetically identical mycelia, even when their original rhythms were different before fusion^39^.

Third, in the baited dishes, significantly large causal relationships were mostly restricted to the electrode pairs that included electrode CH4 (the bait location) as the *Causal* electrode. This suggests that causal relationships in the electrical activities had a potential directionality from electrode CH4 to the other electrodes. This result, combined with the second point discussed above, suggests that the hyphae colonizing the wood bait functioned as a pacemaker of the electrical activities and affected the electrical activities of the entire mycelial body within a dish. Although fungal mycelia do not have a constant “active center” like the heart or brain of mammalian bodies, every part of their hyphal body might be able to act as the temporal center of activity in response to local stimuli, such as resource deposition^39,44^. In slime molds, which have a body system with an uncentralized network similar to that of fungi, local changes in oscillation because of environmental stimuli spread into neighboring parts of the network, thus forming modules of synchronized oscillation rhythms that are ultimately stored as memory of the original stimuli within the network^45^. This process is considered as an important mechanism of learning in slime molds. Similarly, in the mycelial body of cord-forming wood decay fungi, previous studies have suggested that they may have a memory of the direction of resources^6,7^. If the baited parts of the fungal mycelium act as a temporal pacemaker of electrical oscillation and affect other parts of the mycelium, as shown in the present study, this process could be a mechanism of memory and learning in fungal mycelia. Furthermore, the application of artificial electrical fields, that alter or disturb fungal electrical rhythms, may in turn affect fungal wood decay activities^46^.

Fourth, the significance of the causal effects across the electrodes in baited dishes disappeared when a clear and large-magnitude oscillation of electrical potential started at the baited position around day 60 from the start of the experiment. It has been reported that the mycelia of *P. brunnescens* leave their original resource and completely migrate to the new resource within 42 days when the mycelium is energetically starved^7^. Because the present study was conducted on plain agar, the wood bait was almost the only available resource in the system except for some carry-over from the inoculum agar plug at the center, which may be negligible. Therefore, in the present study, it might be possible that the majority of mycelium of *P. brunnescens* had already moved into the wood bait from other parts of the agar medium after 60 days of incubation, although we did not assess the viability of mycelium at the position of every electrode after the experiment. Another possibility is that the mycelium was still viable all over the mycelium on the agar, and the oscillation that started after 60 days of incubation might generate a longer-term causal relationship across the mycelium. In this study, we could not analyze the causal effects for longer than five days because of the data limitation. Monitoring studies with longer oscillation duration without blank periods are required in the future.

In summary, we found significant causal effects in the electrical activities across the mycelium of a cord-forming wood decay basidiomycete, *P. brunnescens*, with potential directionality from the baited location to other parts of the mycelium. This observation was made via long-term (> 100 days) monitoring of electrical potential in the mycelia. We also found stable electrical oscillation in the baited mycelia, and the oscillation period (seven days) was longer than any previously reported oscillatory phenomena in fungi to date. These results indicate the presence of an undiscovered electrical signal transfer and whole-body integration in fungal mycelia, which is important for understanding the flexible and integrated behavior of fungal mycelia^44^.

### Supplementary Information

Supplementary Figures.

## Data Availability

The data presented in this study are available on request from the corresponding author.
