# Supplementary material for: Electrical integrity and week-long oscillation in fungal mycelia
Source: Sci Rep. 2024 Jul 6;14:15601. doi: 10.1038/s41598-024-66223-6 (PMC11227530; doi:10.1038/s41598-024-66223-6)

Fig. S1a Effective transfer entropy (ETE) in the three replicate dishes with a pine wood block (Bait_replication no.) and the three control dishes without wood bait (Cont_replicate no.) during incubation period (0–90 d). The rows in each figure represent the *Causal* electrodes, and the columns represent the *Result* electrodes. A dot indicates the average ETE per day: red, significant ($p < 0.05$); blude, not significant. The causal time delay was set to be ten minutes.

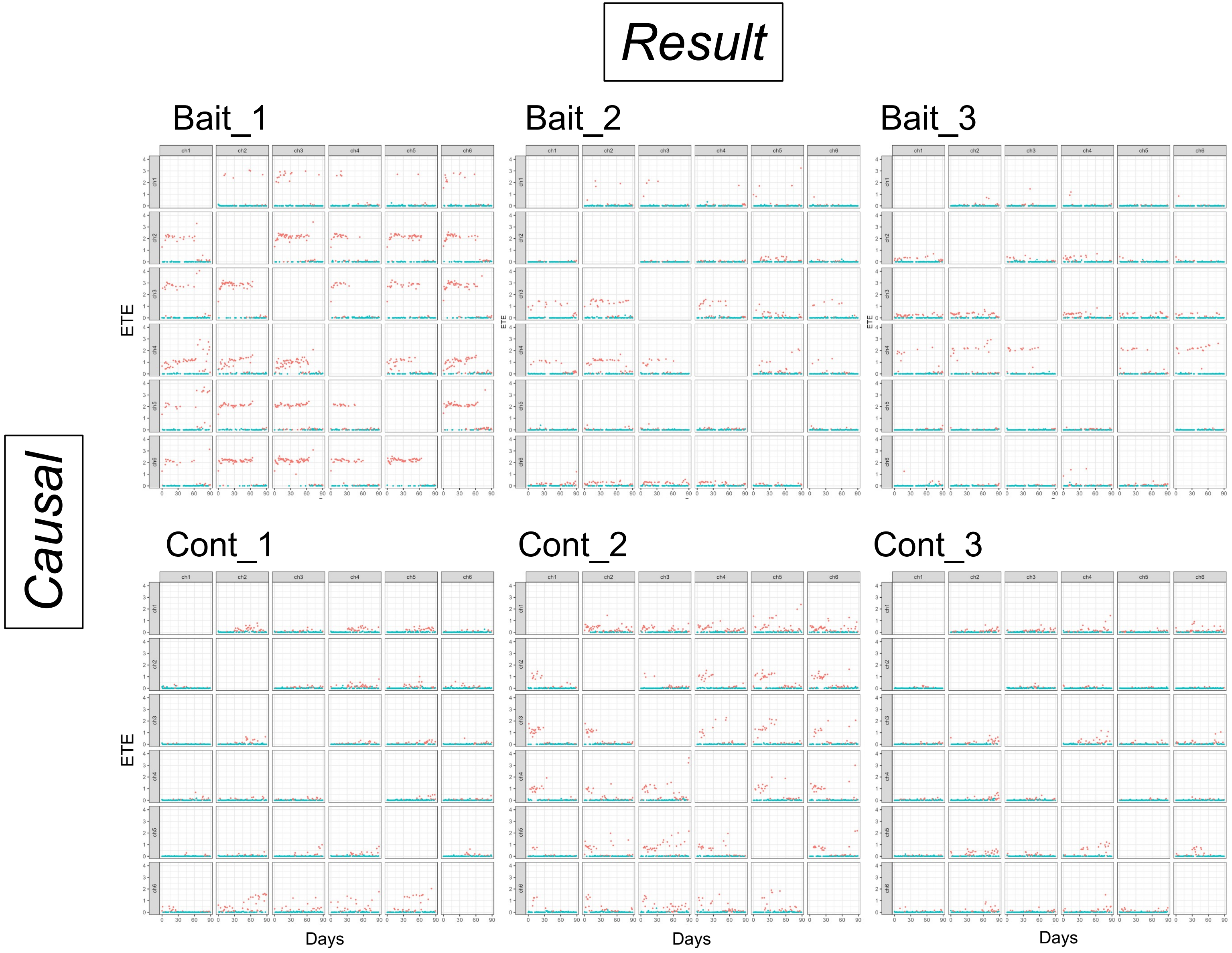

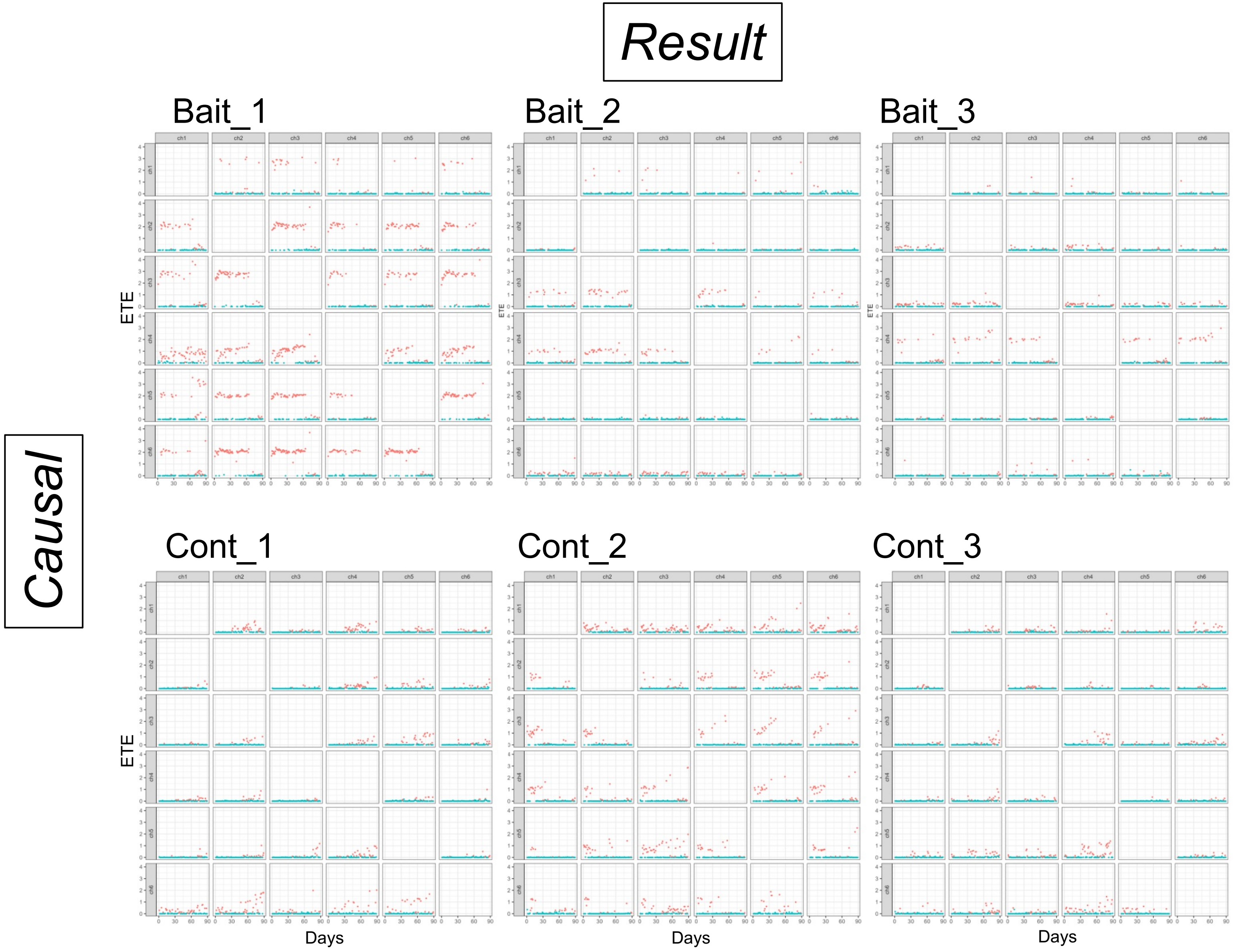

Fig. S1b Effective transfer entropy (ETE) in the three replicate dishes with a pine wood block (Bait_replication no.) and the three control dishes without wood bait (Cont_replicate no.) during incubation period (0–90 d). The rows in each figure represent the *Causal* electrodes, and the columns represent the *Result* electrodes. A dot indicates the average ETE per day: red, significant ($p < 0.05$); blude, not significant. The causal time delay was set to be 720 minutes (12 hours).

Fig. S1c Effective transfer entropy (ETE) in the three replicate dishes with a pine wood block (Bait_replication no.) and the three control dishes without wood bait (Cont_replicate no.) during incubation period (0–90 d). The rows in each figure represent the *Causal* electrodes, and the columns represent the *Result* electrodes. A dot indicates the average ETE per day: red, significant ($p < 0.05$); blude, not significant. The causal time delay was set to be 1440 minutes (one day).

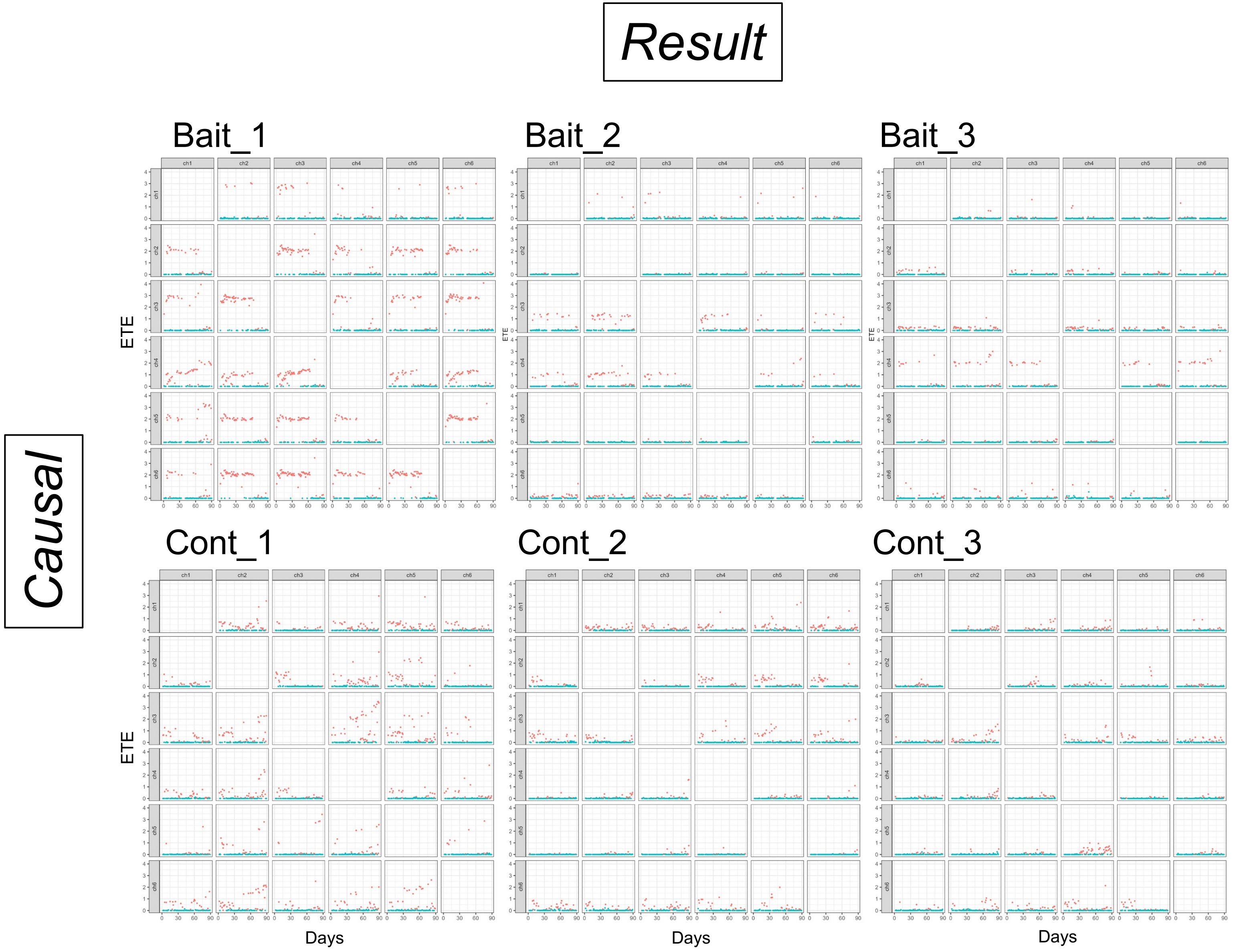

Fig. S1d Effective transfer entropy (ETE) in the three replicate dishes with a pine wood block (Bait_replication no.) and the three control dishes without wood bait (Cont_replicate no.) during incubation period (0–90 d). The rows in each figure represent the *Causal* electrodes, and the columns represent the *Result* electrodes. A dot indicates the average ETE per day: red, significant ($p < 0.05$); blude, not significant. The causal time delay was set to be 7200 minutes (five days).

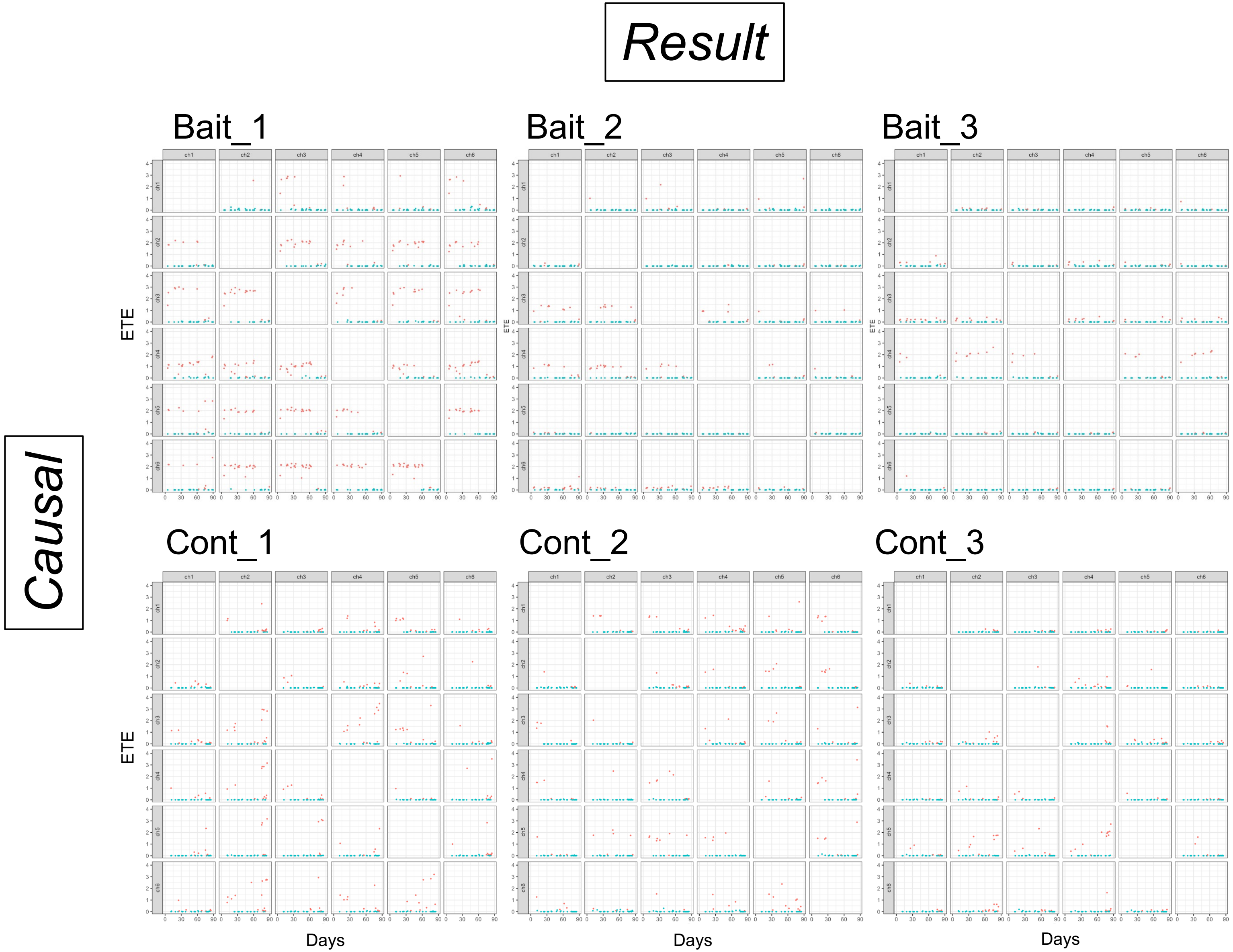

Supplement: Supplementary file 1 — Supplementary Figures. [file 41598_2024_66223_MOESM1_ESM.pdf]
